# Predatory mites double the economic injury level of *Frankliniella occidentalis* in strawberry

**DOI:** 10.1007/s10526-016-9747-y

**Published:** 2016-06-15

**Authors:** Clare Sampson, William D. J. Kirk

**Affiliations:** Centre for Applied Entomology and Parasitology, School of Life Sciences, Keele University, Newcastle Under Lyme, Staffordshire ST5 5BG UK

**Keywords:** Thysanoptera, Thripidae, Western flower thrips, Acari, Phytoseiidae, *Neoseiulus cucumeris*, Economic injury level, IPM

## Abstract

The western flower thrips *Frankliniella occidentalis* (Pergande) (Thysanoptera: Thripidae) causes bronzing to strawberry fruit. Management of insecticide-resistant strains relies on the integration of predators with carefully timed use of the few insecticides available. Effective management requires better understanding of economic injury levels (EILs) and the factors that affect them. The densities of *F. occidentalis* and the predatory mite *Neoseiulus cucumeris* (Oudemans) (Acari: Phytoseiidae) were manipulated in field experiments. All stages of flower and fruit were susceptible to thrips damage, but larvae caused nearly twice as much damage as adults per individual. The EIL was about four adult thrips per flower in the absence of predators, but increased to over eight at densities of *N. cucumeris* typical of good establishment in crops. The EIL could be increased by about 0.7 adult thrips per flower for every *N. cucumeris* per flower. The results were supported by measurements of EILs in commercial crops.

## Introduction

The western flower thrips *Frankliniella occidentalis* (Pergande) (Thysanoptera: Thripidae) is an increasing pest problem in strawberry crops (*Fragaria* × *ananassa* Duchesne ex Rozier) across the world. The use of polythene tunnels for soft-fruit production has favoured the pest’s development and insecticide-resistant strains are widespread (Sparks et al. 2012). In the UK, much strawberry is grown as a semi-protected crop (in open-sided polythene tunnels that are removed in the winter). Losses of 10–15 % due to thrips damage are typical in these crops and some growers have lost entire crops, with the total UK loss estimated at £7–11 million per year (R. Harnden, pers. comm., 2014). Management of insecticide-resistant strains relies on the integration of natural enemies with carefully timed applications of the few insecticides available for use on strawberry (Rahman et al. 2012).

The adoption of thresholds for timing insecticide treatments is likely to reduce the selection pressure for resistance and therefore maintain or improve the efficacy of insecticides (Denholm and Jespersen 1998), as well as reducing the side-effects on natural enemies (Rahman et al. 2011). Most thresholds developed for strawberry crops are based on assessment of thrips density in flowers, as there is a strong correlation between numbers of *F. occidentalis* per flower and fruit damage (Steiner and Goodwin 2005b; Coll et al. 2007; Sampson 2014). Published economic injury levels (EILs) vary considerably for strawberry, from 3 to 24 thrips per flower, according to thrips stage monitored (i.e. adults only or adults and larvae combined) (Steiner and Goodwin 2006), cultivar (Rahman et al. 2010) and sale price (Coll et al. 2007).

There is a need to establish EILs under field conditions in UK crops as there is some evidence that the type and amount of thrips damage may differ with climate. For example, thrips may cause relatively more damage at higher temperatures (Steiner and Goodwin 2005a), and where higher thrips numbers are tolerated, fruit weight is affected as well as bronzing (Coll et al. 2007). In UK crops, unacceptable fruit bronzing usually occurs before fruit weight is affected. Above this threshold of bronzing, the sale price is reduced and at this point the losses from fruit bronzing far exceed the cost of treatment (insecticide or beneficial insect release) (Sampson and Kirk 2013), so we have used the level of bronzing at which fruit gets downgraded to a lower price in commercial pack-houses to define our EIL. The appropriate action when approaching this threshold will vary between farms, but could include further releases of natural enemies.

Where predators are established, EILs can often be increased. In laboratory-based studies, Shakya et al. (2010) estimated that EILs can be increased by about 40 % for each *Orius* bug (Hemiptera: Anthocoridae) per flower and by one or two thrips per flower for each *Neoseiulus cucumeris* (Oudemans) (Acari: Phytoseiidae) per flower, based on predation rates on strawberry. Although *Orius* spp. establish well in southern Europe, the colder temperatures in northern Europe can limit their use, and control there currently relies on *N.* *cucumeris.* Few UK strawberry growers currently take into account the predator numbers when deciding when to treat with insecticide. Further data are required to demonstrate how the presence of mites reduces fruit damage under field conditions in northern Europe.

On strawberry, the most important damage due to *F. occidentalis* is bronzing (russeting) on fruit (Katayama 2005; Steiner and Goodwin 2005a; Coll et al. 2007; Sampson 2014). Small amounts of cosmetic bronzing are sufficient for fruit to be downgraded. Some strawberry cultivars are more tolerant than others to thrips damage (Kitamura and Kashio 2004), and differences could be used to select for resistance to thrips. Other cultivars are more favourable for *F. occidentalis* population growth (Rahman et al. 2010), so the relationship between thrips density and bronzing damage needs to be quantified under local conditions in order to develop EILs. The limited data on the susceptibility of different strawberry fruit stages to bronzing appear contradictory. Steiner and Goodwin (2005a) found bronzing occurred at the young green fruit stage, Coll et al. (2007) found bronzing occurred on mature fruit and Nondillo et al. (2010) found young and mature fruit stages equally susceptible to bronzing. Further data are needed to identify the timing of damage and the thrips stage(s) causing that damage so that growers can take appropriate action.

The aim of this study was to gain a better understanding of economic injury levels (EILs) and the factors that affect them in semi-protected strawberry crops in northern Europe. In particular, to demonstrate in the field the extent to which EILs can be increased in the presence of predatory mites. *N. cucumeris* was investigated as it is the main predator used by growers in this region. EILs were also measured in commercial crops in order to interpret the results.

## Materials and methods

### The effect of* F. occidentalis* on strawberry fruit damage

To test the relationship between thrips density and subsequent fruit bronzing, different numbers of adult female thrips were bagged on individual flowers and the amount of bronzing was recorded subsequently on the fruit that developed from those flowers. The experiment was carried out in an open-sided polythene tunnel (2 m × 5 m), at Keele University, Staffordshire, UK (N 53°00.37′ W 2°27.71′), from late June to early August 2011. Strawberry (*Fragaria* × *ananassa* Driscoll Camarillo) was grown in coir growbags (10 cm wide × 100 cm long, BVB Sublime, Maasland, the Netherlands), each containing ten flowering and fruiting plants. The treatments were 0, 2, 4, 8 or 16 adult female *F. occidentalis* per flower. The experiment was a randomised complete block design, with 17 blocks (growbags) and one replicate per block. Five fully-open flowers, that were similar in size and position on the plant, were selected per block. Each flower was enclosed in a bag made of horticultural fleece (approx. 8 cm × 8 cm), tied to the flower stem with PTFE tape (12 mm wide). In this and subsequent flower-bagging experiments, the flowers had been available to bees for several hours to days before bagging and bees were very active in the tunnel, so they were assumed to have been pollinated.

The *F. occidentalis* for the experiment were reared on potted florists’ chrysanthemum *Chrysanthemum* × *morifolium* Ramat. (Sampson 2014). Microcentrifuge tubes (2 ml) containing the appropriate numbers of adult thrips, or an empty tube for the control, were placed in the flower bags, opened, and left in situ to allow the thrips to move to the flower. After one week the fleece bags were removed from the flowers to allow the fruit to develop normally. All fruit were harvested at the late white-fruit stage (see fruit sampling below). Each fruit was assessed for damage by counting the numbers of seeds surrounded by bronzing. A few fruit that had been partially eaten by other pests (e.g. toads) or affected by powdery mildew were omitted from the analysis. The fruit were weighed and the total numbers of seeds per fruit were counted.

The EIL was calculated from a regression of log_10_ (number of bronzed seeds + 1) (*y*) on log_10_ (thrips per flower + 1) (*x*). Harvested red fruit (Driscoll Camarillo) on a nearby farm was downgraded when there was bronzing around 10 % of the seeds (Sampson and Kirk 2013), so the EIL was calculated by substituting 10 % of the mean seed number for number of bronzed seeds in the regression equation.

### The susceptibility of flower or fruit stages to adult or larval thrips damage

In order to determine the relative susceptibility of different flower and fruit stages to adult or larval thrips, the same numbers of adults or second-instar larvae were bagged for seven days on different stages of flower or fruit, then kept free of thrips by regularly removing any newly arrived thrips from the fruit. The amount of bronzing that developed subsequently on the red fruit was scored. Red fruit was assessed in this experiment so that direct comparison could be made with the fruit that had been infested at the red fruit stage. The experiment was carried out in August 2012, in the Keele University polythene tunnel, using the same growing system, strawberry cultivar (Driscoll Camarillo) and cultural methods described above. The experiment was laid out in a randomised complete block design, with ten blocks (growbags) and one replicate per block (n = ten flowers or fruit). There were 12 treatments: four stages of strawberry (fully-open flowers, green fruit, white fruit and red fruit), each infested with six *F. occidentalis* second-instar larvae, six adult female *F.* *occidentalis* or no thrips (control). Each flower or fruit was enclosed in a bag made of horticultural fleece and thrips were taken from the culture at Keele University, as described above. Any invertebrates observed in the flowers were removed with a damp paint brush before the thrips were released. After one week the fleece bags and all visible thrips were removed with a damp paintbrush and then any further thrips removed every three days until harvest. Fruit damage was assessed on harvested red fruit by counting the number of seeds surrounded by bronzing. A few fruit that had been partially eaten by other pests were excluded from the analysis.

### The effect of* N. cucumeris* on thrips damage to strawberry

In order to test the effect of *N. cucumeris* on fruit bronzing caused by thrips, adult female *F.* *occidentalis* were bagged on individual flowers, with or without the predator *N. cucumeris*, and the amount of bronzing was recorded subsequently on the fruit that developed from those flowers. The experiment was carried out at thrips densities of four and eight per flower, as these spanned the range of densities at which the onset of damage had been observed in the field. Both experiments were carried out in June 2013 using the same polythene tunnel, strawberry cultivar (Driscoll Camarillo) and growing methods (growbags) described above.

The two treatments were with and without *N. cucumeris*. The experiment was laid out in four blocks, with each block consisting of a growbag with predators and a growbag without. Two flowers on separate randomly selected plants were used on each growbag. Each growbag was surrounded by blue sticky traps on the ground in order to prevent the spread of predatory mites from treated to untreated growbags. Fully-open flowers of a similar size were selected, and then each was infested with four adult female *F. occidentalis*, and enclosed in a nylon-mesh pyramid teabag (75 mm long × 33 mm wide at the top, Tea Forte, St Albans, UK), which was cut along one side to allow access and then sealed with clear adhesive tape. The mesh was fine enough to prevent thrips or mite escape. Treated flowers were infested at the same time with five commercial, active *N. cucumeris* (Ambsure, BCP Certis, Ashford, UK), which is equivalent to a good establishment of the predatory mites in the field (Rahman et al. 2012). Typical release rates for thrips in the UK are about 25 *N. cucumeris* per plant repeated regularly (e.g. fortnightly) or as required (Sampson, unpublished data, 2014). Mixed-aged and mixed-sex *N. cucumeris* were used to match what growers use. Tubes containing the predators, or an empty tube for the control, were placed in the flower bags, opened, and left in situ to allow the mites to move to the flower. After one week, the flower bags were removed to allow the fruit to develop normally. All fruit were harvested at the late white-fruit stage, 27 days after they were infested (see fruit sampling below). Each fruit was assessed for damage by counting the number of seeds surrounded by bronzing. Each fruit was weighed and the total number of seeds per fruit was counted. The experiment was repeated with eight adult female *F. occidentalis* per flower instead of four and harvested at the late white-fruit stage, 30 days after they were infested.

### Economic injury levels in commercial crops

Six fields were sampled monthly from May to September during the 2012 growing season within a 40 km radius from N 52°37′ W 1°45′. In each field, the plots were spaced regularly across the field and there were either ten plots of 2 m^2^ (ten plants sampled per plot) or eight plots of 10 m^2^ (40 plants sampled per plot). Thus a total of 100–320 plants were sampled per field on each occasion. One medium-aged flower and one late white fruit were sampled per plant (see fruit sampling below). The numbers of adult thrips per flower and the numbers of seeds surrounded by bronzing per fruit were counted by eye in the field using a ×7 head lens (optiVISOR, LightCraft, London, UK). We used counts of adult thrips, rather than adults and larvae, because this is more practical for growers and the assessment of larvae by eye in the field is unreliable (Gonzalez-Zamora and Garcia-Mari 2003). Medium-aged flowers that were fully open, pollen shed and towards the top of the plants were selected in order to obtain a consistent sample. These flowers have the most thrips and most risk of damage (Sampson and Kirk 2012). The number of predatory anthocorid bugs per flower or fruit was also counted. Flowers were pooled and placed in 70 % alcohol so that thrips could be extracted and identified to species. The predatory mite *N. cucumeris* was released by the growers in all the fields sampled, but with varying amounts and frequencies. In addition to the fruit damage assessments, each fruit was picked and examined by eye, using a ×7 head lens and the presence or absence of *Neoseiulus* was recorded per fruit.

EILs were calculated as above, except that the mean number of bronzed seeds and the mean number of thrips per flower from each plot on each occasion were used as points in the regression. Fruit bronzing on a particular date was regressed on thrips density on the same date, as this gave at least as strong a correlation as when damage was measured at various time intervals after measurement of thrips density (Sampson 2014).

### Fruit sampling

With the exception of the experiment on the stages of flower and fruit damaged by thrips, fruit damage was assessed at the late white-fruit stage using  ×7 head lens. This was when fruit was fully swollen and turning pink, at the end of growth stage 85 (Meier et al. 1994), a few days before turning fully red. Red fruit could not be sampled in commercial crops because it was not always available following picking and the selective picking of undamaged red fruit would have biased our samples. The late white-fruit stage was the nearest stage to red that could always be sampled reliably and without bias. Use of this stage allowed consistency across experiments. However, damage can sometimes be less obvious on red fruit than on late white fruit (Sampson and Kirk 2013). A bright light and a head lens were used to assess damage to red fruit in the pack-house and this would have mitigated against this effect. However, the EILs in this paper may be slightly on the conservative side (lower) as a result.

### Statistical analysis

The experimental data were analysed by one-way or two-way analysis of variance, as appropriate. Fruit bronzing, fruit weight and seeds per fruit, were compared between the treatments, which included number of thrips per flower, flower and fruit stage, thrips stage and presence of predatory mites, depending on the experiment. All data were transformed to log_10_ (*x* + 1) to homogenise the variance. Residuals were checked for normality. Multiple comparisons used Tukey’s test. Tables and figures show untransformed means for adult thrips per flower and seeds per fruit to allow more intuitive comparison with counts that would be used by growers. Statistical analysis was carried out with Minitab 16 (Minitab Inc., USA).

## Results

### The effect of *F. occidentalis* on strawberry fruit damage

**Fig. 1:**
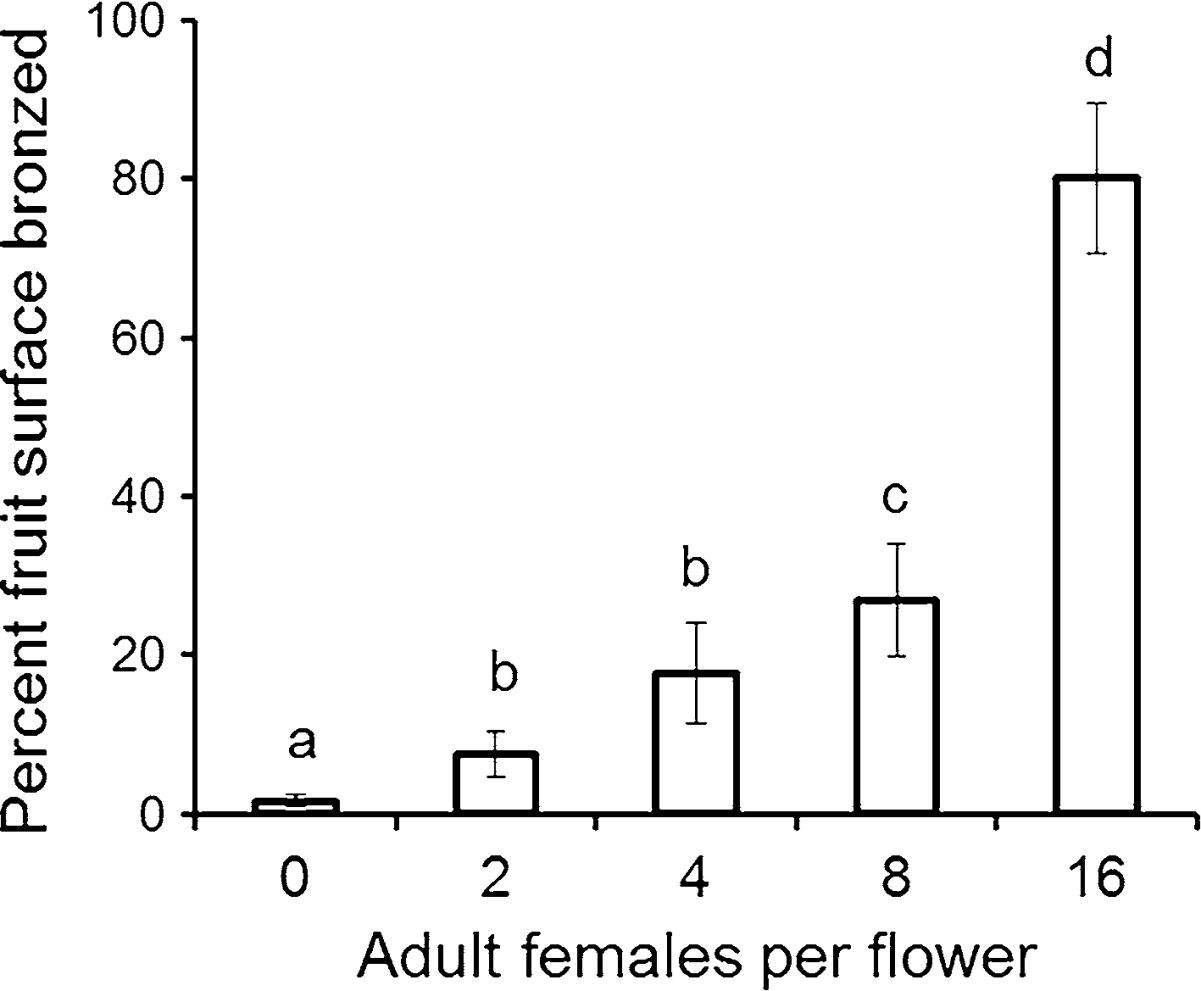
The mean percentage (±SE) of seeds surrounded by bronzing per fruit following infestation of strawberry flowers with different numbers of adult *F. occidentalis* in a semi-protected strawberry crop (n = 16–17 flowers). Means with the same letter are not significantly different (Tukey’s test, *P* > 0.05). Untransformed data are presented, whereas the analysis was of log-transformed data

When thrips were confined on flowers, fruit bronzing increased with the number of adult female *F. occidentalis* per flower (*F*_(4, 63)_ = 51.9, *P* < 0.001, Fig. 1). At low thrips densities (two per flower), there was slight brown tracking under the calyx, with about 8 % of the white fruit surface bronzed. At medium thrips density (four to eight per flower), bronzing covered 18–27 % of the white-fruit surface. At high thrips density (16 per flower) about 80 % of the white fruit surface was bronzed. No fruit malformation (‘cat-facing’) was found. Very low levels of damage were observed in the controls and this may have occurred because a few thrips were able to move onto control fruit once the bags had been removed. Fruit weight averaged 7.2 ± 0.5 g with no significant difference in weight observed between treatments (*F*_(4, 63)_ = 1.02, *P* = 0.41). The mean number of seeds per fruit was 291 ± 15 seeds.

The regression equation to calculate the EIL was: log_10_ (number of bronzed seeds + 1) = 0.31 + 1.56 log_10_ (thrips density + 1) and was highly significant (*F*_(182)_ = 144, *P* < 0.001; *R* ^2^ = 64 %). The calculated EIL was 4.6 thrips per flower (at 29 seeds bronzed), which was in the absence of predators. The addition of further data for four and eight thrips per flower from the experiment with and without *N. cucumeris* described below gave a similar value of 4.1 thrips per flower.

### The susceptibility of flower and fruit stages to adult and larval thrips damage

**Fig. 2:**
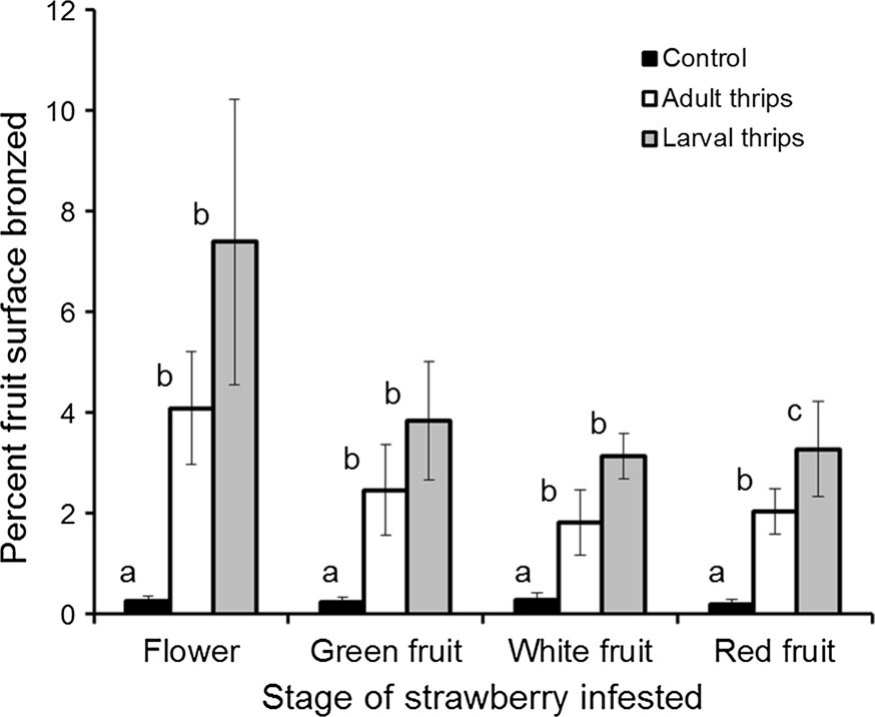
The mean percentage (±SE) of seeds surrounded by bronzing per red fruit following infestation of strawberry flowers, green fruit, white fruit or red fruit for seven days, with six adult female, or six second-instar larval *F. occidentalis*, or an untreated control (n = 7–10 flowers or fruit). Larvae and adults caused more damage than the controls (F_(2,100)_ = 77.6, *P* < 0.001) and larvae caused more damage than adults overall (Tukey’s test, *P* = 0.03), but there was no significant difference in damage between the strawberry stages (F_(3,100)_ = 1.5, *P* = 0.23). Multiple comparisons are shown within each flower or fruit stage, where means with the same letter for the same stage are not significantly different (Tukey’s test, *P* > 0.05). Untransformed data are presented, whereas the analysis was of log-transformed data

Both adult and larval *F. occidentalis* caused damage to all stages of strawberry tested (Fig. 2). There was a trend towards more damage when flowers were infested rather than fruit stages, although the overall differences were not statistically significant (*F*_(3,100)_ = 1.5, *P* = 0.23) and there was no significant interaction for strawberry stage × thrips treatment (*F*_(6,100)_ = 0.5, *P* = 0.78). There was a significant difference in the amount of damage between thrips larvae, thrips adults and control treatments (*F*_(2,100)_ = 77.6, *P* < 0.001). Larvae caused nearly twice (×1.7) as much damage as adults over seven days (Tukey’s test, *P* = 0.03), and larvae and adults both caused more damage than controls (Tukey’s test, *P* < 0.001), with mean bronzing (number of seeds) across all strawberry stages of 15.0 ± 2.6, 8.9 ± 1.4 and 0.9 ± 0.2 for six larvae, six adults and no thrips respectively.

### The effect of *N. cucumeris* on thrips damage to strawberry

**Fig. 3:**
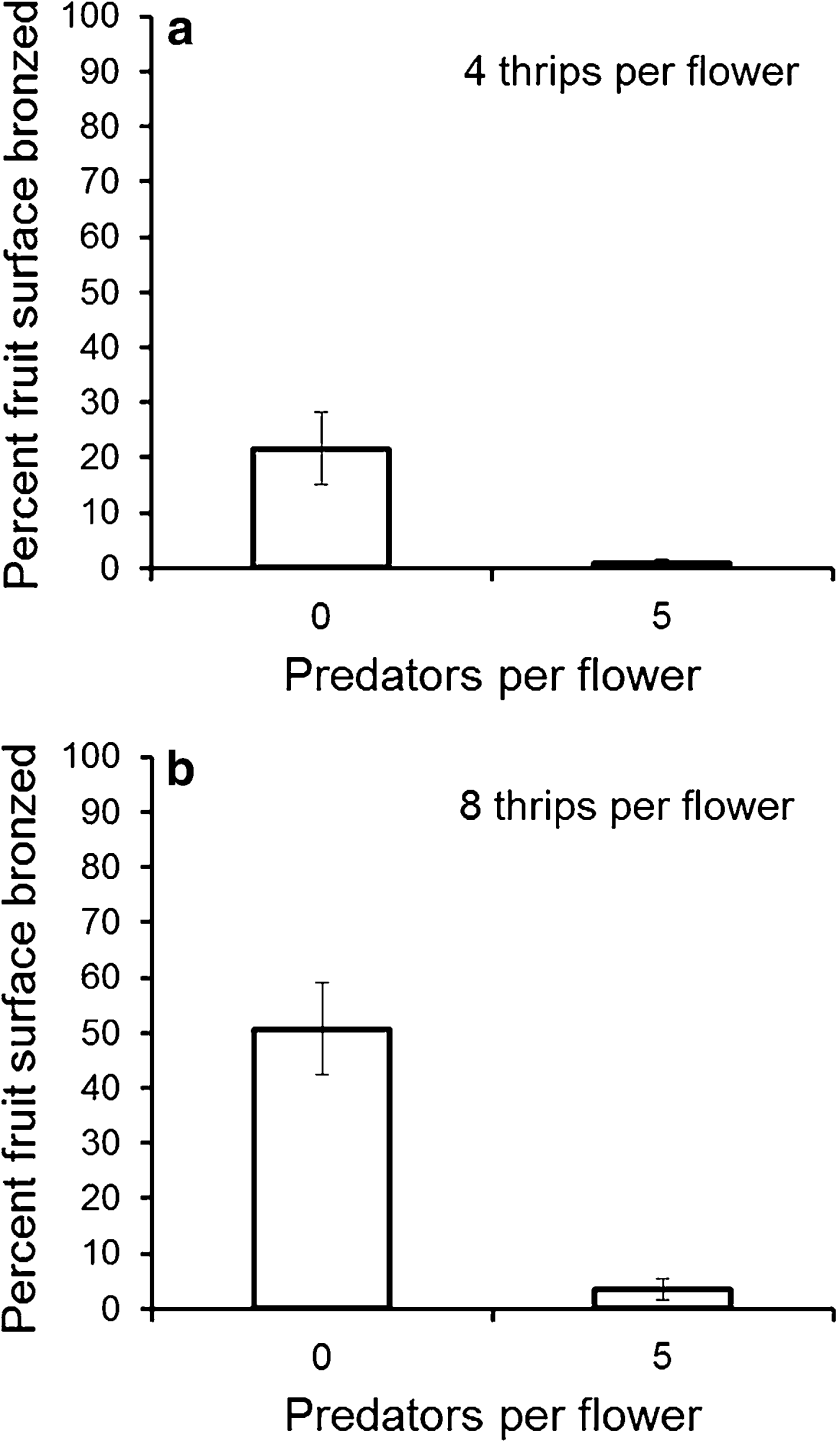
The mean percentage (±SE) of seeds surrounded by bronzing per fruit following infestation of strawberry flowers with **a** four adult female *F. occidentalis* per flower and **b** eight adult female *F.* *occidentalis* per flower, with or without the predator *N. cucumeris* (n = eight flowers). The means are significantly different in (**a**) (*F*_(1, 14)_ = 44.6, *P* < 0.001) and in (**b**) (*F*_(1, 14)_ = 32.2, *P* < 0.001). Untransformed data are presented, whereas the analysis was of log-transformed data

The addition of *N. cucumeris* (five per flower) to flowers containing four adult female *F.* *occidentalis* prevented nearly all fruit bronzing (*F*_(1,14)_ = 44.6, *P* < 0.001, Fig. 3a). There was no significant difference in fruit weight between treatments (*F*_(1,14)_ = 0.11, *P* = 0.74), which averaged 4.5 ± 0.7 and 4.3 ± 1.1 g with and without *N. cucumeris* respectively, or number of seeds per fruit (*F*_(1,14)_ = 2.5, *P* = 0.14), which averaged 314 ± 17 and 351 ± 22 with and without *N. cucumeris* respectively.

The addition of *N. cucumeris* (five per flower) to flowers containing eight adult female *F.* *occidentalis* significantly reduced fruit bronzing (*F*_(1,14)_ = 32.2, *P* < 0.001, Fig. 3b). There was no significant difference between treatments in fruit weight (*F*_(1,14)_ = 0.16, *P* = 0.69), which averaged 4.6 ± 0.3 and 4.8 ± 0.5 g with and without *N. cucumeris* respectively, or number of seeds per fruit (*F*_(1,14)_ = 0.5, *P* = 0.51), which averaged 353 ± 9 and 361 ± 7 with and without *N. cucumeris* respectively.

The amount of bronzing exceeded the critical threshold of 10 % of seeds (i.e. about 35) for both four and eight thrips per flower in the absence of predatory mites, indicating that the EIL was under four thrips per flower. In contrast, in the presence of five predatory mites, the critical threshold was not exceeded at either four or eight thrips per flower, indicating an EIL of over eight thrips per flower. The EIL was at least doubled by a density of *N. cucumeris* equivalent to good establishment in the field. Five *N. cucumeris* prevented nearly all the damage that would have been caused by eight thrips in a flower (Fig. 3b), thus the EIL was increased from 4.6 or less to 8.0 thrips per flower, which is about 0.7 adult thrips per flower for every *N. cucumeris* per flower.

### Economic injury levels in commercial crops

EILs of between six and 11 adult thrips per flower were measured in four commercial crops (Table 1). No economic crop loss due to thrips damage was observed in two crops where thrips numbers remained below four adults per flower throughout the season with the presence of predatory mites (Table 1). The lowest EIL (6.3 adult thrips per flower) was observed in the field with the lowest cover of predatory mites, and the highest EIL (10.6 adult thrips per flower) was in the field with the highest cover of predatory mites (Table 1). These EILs are similar to those observed in the experiments with and without *N. cucumeris*, but cannot be quantified further as the numbers of predatory mites per fruit were not counted. The *Neoseiulus* were predominantly *N. cucumeris* with a small percentage of *N. californicus* (McGregor). Predatory anthocorid bugs, *Anthocoris nemorum* (L.)*, Orius niger* (Wolff) and *Orius laevigatus* (Fieber), were present in very low numbers, occupying <1 % of flowers and fruit. Over 80 %, and typically over 95 %, of adult thrips in the fields were *F. occidentalis* at times when economic fruit damage was observed (*n* > 2000). The other thrips species present were mainly *Thrips major* Uzel.

**Table 1:** Economic injury levels (EIL) in six commercial semi-protected strawberry fields in 2012

Site	Cultivar	Peak thrips density (mean ± SE)^a^	% fruit with predatory mites ± SE^b^	EIL (adult thrips per flower ± SE)	*R* ^2^ (%)	*P*
Field1	Camarillo	6.0 ± 0.5	5 ± 2	6.3 ± 0.4	88	<0.001
Field2	Finesse	12.5 ± 0.8	62 ± 5	8.7 ± 1.3	65	0.009
Field3	Camarillo	18.5 ± 0.8	60 ± 3	8.8 ± 0.4	63	<0.001
Field4	Camarillo	17.1 ± 0.9	72 ± 3	10.6 ± 1.1	62	<0.001
Field5	Camarillo	1.0 ± 0.2	72 ± 4	No economic damage^c^		
Field6	Finesse	3.1 ± 0.4	65 ± 3	No economic damage^c^		

Each was sampled monthly from May to September, with 100–320 plants sampled on each occasion. The EILs were calculated from regressions of fruit bronzing on thrips density for each site (see the materials and methods section). The *R* ^2^ goodness of fit and *P* value for each regression are also given. The growers applied one or two spinosad (Tracer) treatments for thrips during the whole season, whereas weekly or fortnightly treatments with various active ingredients were used in the years before natural enemies were used

^a^The highest mean number of adult thrips per flower recorded in the monthly samples

^b^In the sample with the peak thrips density

^c^No fruit was downgraded due to thrips damage throughout the season

## Discussion

No fruit malformation was found in the experiments in which flowers and fruit were caged with thrips, even when mean bronzing reached 80 %. This is consistent with other recent studies (Easterbrook 2000; Nondillo et al. 2010). Earlier reports of fruit malformation by thrips may have been reporting capsid damage (Buxton and Easterbrook 1988).

*Frankliniella occidentalis* larvae caused more damage per individual than adults (Fig. 2), as found in other crops, such as nectarines (Pearsall 2000), presumably because they feed more. The large reduction in damage (>90 %) in the presence of *N. cucumeris* (Fig. 3), which feed only on thrips larvae (Jacobson et al. 2001), provided evidence that it is the larvae that cause the majority of the damage. It should be emphasised that although the numbers of adult thrips per flower correlated well with thrips damage in our studies and elsewhere (Steiner and Goodwin 2005a), it is the larval progeny from the adults in the flower that are doing most of the fruit damage and it is these larvae, not the adults, that are being eaten by *N. cucumeris*. Higher numbers of adults than were used here could produce enough damage by themselves for fruit downgrading, in which case predatory mites would not be able to prevent it.

The field experiments demonstrated an EIL of about four adult thrips per flower in the absence of predators, but this doubled to over eight thrips per flower in the presence of five predatory mites per flower. These figures were consistent with the data from commercial crops where crops with good mite establishment had EILs of 8.7–10.6 and one crop with poor mite establishment had an EIL of 6.3 (Table 1). The EILs from the experiments and commercial crops were broadly similar to those identified in Australia (Steiner and Goodwin 2005a), France (Grasselly 1995) and Israel (Shakya et al. 2010). Higher EILs are likely in regions where there are typically more naturally occurring and released anthocorids (Shakya et al. 2010), which are more voracious thrips predators than mites. In contrast, anthocorids were present in <1 % of flowers in this northern European study.

The reduction in strawberry fruit bronzing resulting from *N. cucumeris* (Fig. 3) matches that predicted by Shakya et al. (2010), based on *N. cucumeris* predation rates on thrips larvae. The predatory mites prevented nearly all damage at an initial thrips density of four adult female thrips per flower (Fig. 3a) by reducing the more damaging larval progeny, demonstrating that low levels of thrips can be managed with predatory mites. Shakya et al. (2010) estimated that the EIL could be increased by one or two motile thrips (adults and larvae), for each predatory mite present per sampling unit, which is similar to this study, in which the EIL could be increased by 0.7 adult thrips per flower per predatory mite per flower. The data from commercial crops support the experimental studies by showing increased EILs with increasing *N. cucumeris* establishment (Table 1).

Decision making needs to take predator establishment into account. This can reduce the number of insecticide applications and thus reduce the side-effects on natural enemies and slow the development of insecticide resistance by the thrips. Reduced spraying also increases the number of residue-free crops, increases the productivity and marketability of the fruit and can benefit pollinators.

Control of *F.* *occidentalis* using *N. cucumeris* is a preventative treatment that relies on good distribution of the predatory mites over the crop before adult thrips populations build up (Fitzgerald and Jay 2013), yet the distribution of mites was observed to be patchy in the fields sampled in this study (Table 1). Further data are required to determine the numbers or percentage cover of predators needed to reduce strawberry fruit damage in the field and to determine a reliable monitoring method for growers. Since *N. cucumeris* are found under the calyx of strawberry fruit and can be seen using a good hand lens in the field (Sampson 2014), growers could perhaps use this simple monitoring method with some training on where to look and how to recognise predatory mites. Improved application methods are required to improve the distribution of the predatory mites in the field and action thresholds are needed to decide when further predators should be released.
